# Variability in quantitative outcomes of instrumental swallowing assessments in adults: a scoping review

**DOI:** 10.1590/2317-1782/20242024046en

**Published:** 2024-09-13

**Authors:** Jayne de Freitas Bandeira, Desiré Dominique Diniz de Magalhães, Leandro Pernambuco

**Affiliations:** 1 Universidade Federal da Paraíba – UFPB - João Pessoa (PB), Brasil.; 2 Universidade Federal de Pernambuco – UFPE - Recife (PE), Brasil.

**Keywords:** Swallowing, Swallowing Disorders, Biological Variation Population, Health Evaluation, Diagnosis, Diagnostic Techniques and Procedures

## Abstract

**Purpose:**

To map scientific evidence on the variability of quantitative parameters extracted by instrumental swallowing assessment tests in adults, using the coefficient of variation.

**Research strategies:**

The methodological procedures recommended by the Joanna Briggs Institute and the extension for scoping reviews of the Preferred Reporting Items for Systematic Reviews and Meta-Analysis (PRISMA-ScR) were followed.

**Selection criteria:**

The search was carried out in the Pubmed/Medline, Lilacs, Cochrane Library, Embase, Web of Science, Scopus and CINAHL databases, as well as in Google Scholar to consult the gray literature.

**Data analysis:**

Two blind and independent reviewers screened the articles by title and abstract. Subsequently, the articles were read in full and selected according to the eligibility criteria. Data were extracted according to a standardized instrument.

**Results:**

363 studies were found, 13 of which were eligible. Most studies had a sample size of less than 30 participants and were made up of healthy individuals. The instrumental exams used were diverse: videofluoroscopy, electrical impedance tomography, laryngeal sensors, high-resolution manometry and surface electromyography. The studies searched for intra-individual variability and the coefficient of variation ranged from low to high variability, as the instruments, parameters and collection procedures were very heterogeneous and non-standardized.

**Conclusion:**

Intra-individual variability of the quantitative outcomes of instrumental swallowing assessments in adults ranged from low to high according to the exam, outcome, presence or absence of underlying disease, consistency and volume of the bolus.

## INTRODUCTION

Swallowing is a complex function that involves multiple structures and neuromuscular regions^(1,2)^. The initial clinical evaluation consists of structural and functional observation to collect data, observe signs and symptoms, and guide clinical reasoning^(3)^. However, in isolation, it may be inefficient to detect non-visible changes that characterize a swallowing disorder^(4)^, compromising the effectiveness of treatment^(5)^.

Therefore, instrumental examinations are important to investigate these parameters more precisely^(6)^. Structural details are analyzed by observing the images from these examinations, obtaining more quantitative data – which have been gaining prominence in light of technological advances^(7)^ because they aid in the diagnostic process^(5,8)^.

In clinical practice, the videofluoroscopy swallowing study (VFSS) provides real-time visualization of food transport through sequential video radiographic images^(9)^. It is considered the reference procedure for evaluating and identifying the risk or occurrence of food penetration or aspiration^(9-12)^, identifying swallowing disorders, and analyzing the effects of treatment strategies^(13-15)^.

Videoendoscopy is also used to detect changes in swallowing. This exam provides visualization of the hypopharynx and larynx, thus enabling the observation of residues, penetration, and aspiration in the laryngopharyngeal region^(15)^. It can be performed in both children and adults^(16)^ and is feasible for quantitative analysis of the duration of the pharyngeal phase^(10)^.

Ultrasonography, in turn, has been used as a complement in the investigation and monitoring of morphometric and kinematic parameters of the oral and pharyngeal phases of swallowing^(17,18)^. Its applicability in the face of technical innovations helps diagnose and treat dysphagia, enabling, for instance, evaluations of hyolaryngeal and tongue movement^(19,20)^. There are also exams such as high-resolution manometry that can reveal changes in pressure and time measurements in the pharynx and esophagus during swallowing, even if there are no complaints or apparent clinical signs^(6)^.

Due to the complexity of extracting and analyzing quantitative measures, most exams require trained and experienced evaluators^(21-23)^ because it is difficult to standardize methods. Moreover, there are intrarater and interrater particularities that make it impossible to compare studies. However, these exams make it possible to investigate aspects related to swallowing performance through images and quantify data^(8)^. Thus, quantification enables the comparison and monitoring of the patient's evolution, providing complementary and guiding information for the therapeutic process^(24)^.

The reliability analysis and validity of assessment instruments in the literature are extremely important to verify the quality of diagnostic information^(25)^. Also, the variability analysis of quantitative data extracted by these tests provides an important contribution. Variability or dispersion is normally investigated using indicators such as the coefficient of variation (CV), calculated by the ratio between the standard deviation and the mean of the data set^(26)^.

CV is a dimensionless measure of variability; therefore, it compares data with different units, and the result is given in percentages^(27)^. Normally, the CV of biological systems ranges from 10-15%^(28)^; when the CV is above 30%, it indicates that the measure has high heterogeneity^(29)^. Thus, the lower the CV, the lower the degree of variability^(30)^.

Dispersion can be influenced by various biological and assessment factors. For instance, instrumental swallowing assessment patterns may differ in the time interval between offers, food consistency, volume, and sequence of attempts^(31)^. By analyzing the variability of the measures, one can understand their homogeneity and representativeness, which helps to identify inconsistencies^(32)^. Thus, it can be analyzed whether these measures actually help to characterize the parameter of interest and thus decide whether they are safe to be applied in practice.

Hence, this review aimed to map the scientific evidence on the variability of quantitative parameters extracted by instrumental swallowing exams through the investigation of the CV. The study was guided by the following research question: “What is the level of variability of quantitative parameters of instrumental swallowing assessments in adults?”.

## METHOD

This scoping review was conducted in accordance with the methodological recommendations of the Joanna Briggs Institute (JBI) for the type of study in question^(33)^ and followed the criteria of the Preferred Reporting Items for Systematic Reviews and Meta-Analysis – Extension for Scoping Reviews (PRISMA-ScR)^(34)^. The protocol for this review was previously published and reported the objectives, eligibility criteria, sources, search strategy, selection, analysis, and data presentation methods^(35)^. It was registered in the Open Science Framework on January 28, 2023 (https://osf.io/p3g2e/). The PCC acronym was used to define the research question, as follows: Population (young and/or older adults who underwent swallowing assessment), Concept (level of variability of quantitative data resulting from the swallowing assessment), and Context (studies using instrumental exams with quantitative results of the CV for the analysis of swallowing parameters).

### Search Strategies

The bibliography was surveyed on December 3, 2022, and an updated search was performed on November 30, 2023, in the databases of PubMed/MEDLINE, LILACS via BVS, Cochrane Library, EMBASE, Web of Science, Scopus, and CINAHL via EBSCO. The gray literature was also searched in Google Scholar, considering only the first 100 articles retrieved in the search. It was not possible to perform the search in the ProQuest database as provided for in the protocol^(35)^ since the authors' institution was not granted access at the time of the search.

The search strategy was based on the combination of descriptors and keywords (Chart 1) adapted for each database. The references in retrieved articles were also considered and manually checked to identify studies that could be relevant to the topic of interest.

**Chart 1:** Search strategies per database

**Database**	**Descriptors**	**Records found December 3, 2022**	**Records found November 30, 2023**
**PubMed/MEDLINE**	(((((((((deglutition disorders[MeSH Terms]) OR (dysphagia[MeSH Terms])) OR (swallowing disorders[MeSH Terms])) OR (deglutition[MeSH Terms])) OR (swallowing[MeSH Terms])) OR (dysphagia)) OR (deglutit*)) OR (swallow*)) AND (“coefficient of variation” [All Fields]))	56	57
**LILACS via BVS**	(“deglutition disorders” OR “dysphagia” OR “swallowing disorder” OR swallow* OR deglutit*) AND (“coefficient of variation”)	4	4
**Cochrane Library**	Dysphagia AND coefficient of variation	3	4
**EMBASE**	('swallowing'/exp OR swallowing OR 'dysphagia'/exp OR dysphagia) AND ('coefficient of variation'/exp OR 'coefficient of variation')	63	64
**Web of Science**	(TS=((“deglutition” OR “deglutitions” OR “swallowing” OR “swallowings” OR “swallow” OR “swallows” OR “deglutition disorders” OR “deglutition disorder” OR “swallowing disorder” OR “swallowing disorders” OR “dysphagia”) AND (“coefficient of variation”)))	46	46
**Scopus**	(TITLE-ABS-KEY((“Dysphagia” OR “Swallowing disorder” OR “Deglutition” OR”Deglutition Disorders”OR “Swallowing”)) AND TITLE-ABS-KEY((“coefficient of variation”)))	61	62
**CINAHL via EBSCO**	Dysphagia OR Swallowing disorders OR Deglutition OR Swallowing OR Deglutition Disorders AND coefficient of variation	30 Full text	40 Full text
**Google Scholar**	(“Dysphagia” OR “Swallowing disorders”) AND (“coefficient of variation”)	2160 Considering only the first 100 studies	2440 Considering only the first 100 studies
**TOTAL**		363	377

### Study Selection

After the search, the study had the following stages:

1. The articles recruited in each database were imported into EndNote software (Clarivate Analytics, PA, USA) for management and removal of duplicates;
2. Two reviewers used Rayyan software (Qatar Computing Research Institute, Doha, Qatar) to blindly screen articles by title and abstract;
3. Conflicts were resolved through analysis by a third reviewer.

The study included all articles available in full in the established sources of evidence, without restrictions on year or language. It excluded studies that did not analyze the level of variability through CV, that did not report the mean and standard deviation to enable the calculation of CV by the researchers, that used alternative formulas to calculate CV, that used instrumental examinations but did not present quantitative results, that evaluated only esophageal swallowing, and that approached only children.

### Data extraction and presentation

The studies were selected for inclusion based on the steps presented in the flowchart recommended by PRISMA-ScR^(34)^. After the initial filters, the two reviewers analyzed the included articles by reading their full text, considering the eligibility criteria to maintain them in the final result. The data were extracted according to the research objectives, following an extraction matrix previously presented in the protocol of this review^(35)^.

## RESULTS

**Figure 1:**
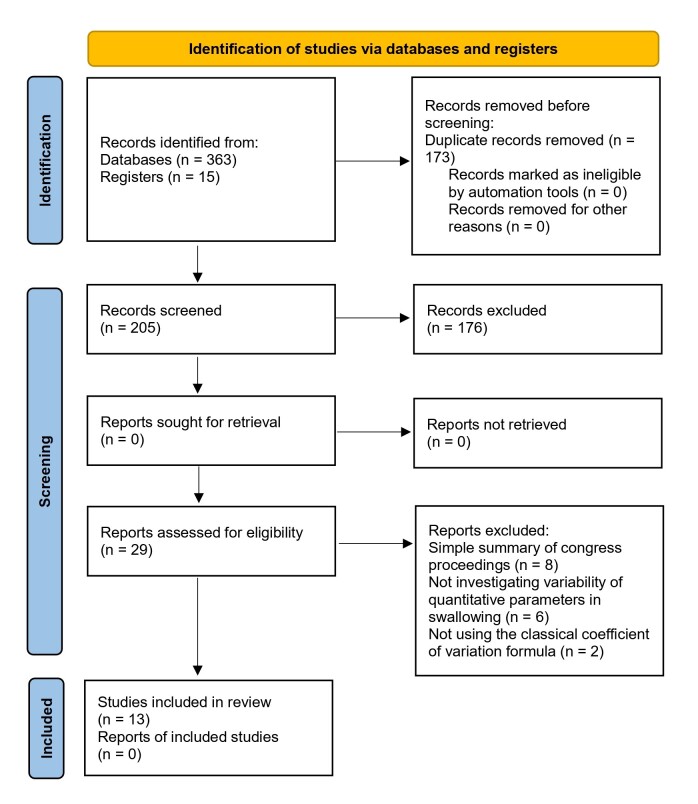
Flowchart of the study selection process – PRISMA-ScR (2020)

The search and selection process found 363 studies, of which 13 were eligible^(6,24,26,30,31,36-43)^ according to the established inclusion criteria (Figure 1).

The data extracted from the articles included in this review are shown in Chart 2. The studies were published between 1990 and 2022, and six of them^(6,24,26,36,37,43)^ were developed in the United States. Six (46.15%) studies^(30,31,36,37,39,40)^ had a sample of fewer than 30 participants. More than 50% of the studies had a mixed population in terms of sex (men and women) and age (young and older adults). Two articles (15.3%) mentioned pairing the sample by sex^(6,43)^.

**Chart 2:** Analysis matrix of studies that used the coefficient of variation to investigate the variability of quantitative parameters or outcomes extracted by instrumental swallowing examinations

**Author, Year, Country**	**Study objective**	**Sample size, Age range, Diagnosis**	**Examination**	**Parameters investigated**	**Utensil, Volume, Consistency offered**	**Coefficient of variation (CV)**
Lof and Robbins^(36)^, 1990, USA	To determine whether temporal swallowing parameters in healthy subjects remain stable in test-retest.	Sample size:	Videofluoroscopy	Oral transit duration (OTD);	Utensil: spoon	OTD = 50% (Lq), 43% (Ss);
		8 women and 8 men		Transition stage duration (TSD);	Consistency: liquid (Lq) and semi-solid (Ss) barium	TSD = 1.14% (Lq), 17.67% (Ss);
		Age range:		Pharyngeal transit duration (PTD);	Volume: 3x 2 ml of each consistency	PRD = 21% (Lq), 36% (Ss);
		group 1: 43-45 years		Pharyngeal response duration (PRD);		PTD = 18% (Lq), 59% (Ss);
		group 2: 63-67 years		Palate velum excursion duration (DOVE);		DOVE =28% (Lq), 41% (Ss);
		Diagnosis: healthy		Duration of maximum hyoid elevation (DOHME);		DOHME = 48% (Lq), 63% (Ss);
				Duration of maximum hyoid anteriorization (DOHMA);		DOHMA = 45 (Lq), 38% (Ss);
				Duration of upper esophageal sphincter opening (DOOUES);		DTOUES = 38% (Lq), 38% (Ss);
				Duration to upper esophageal sphincter opening (DTOUES).		DOOUES = 50% (Lq), 43% (Ss).
Takahashi et al.^(37)^, 1994, USA	To investigate the symmetry and reproducibility of swallowing sounds detected bilaterally in healthy individuals.	Sample size: 5 women and 5 men	Sonography and accelerometers	Duration of sounds (in seconds);	Utensil: NR	Sound duration:
		Age range:		Signal-to-noise ratio (in decibels);	Consistency: liquid (water)	Left: 18.7%-61.4% (mean: 34.0%);
		men - 35.4 years (mean)		Peak 1 of the acoustic signal (< 110 hertz);	Volume: 30x 5 ml	Right: 17.9%-66.3% (mean: 35.3%);
		women - 29.4 years (mean)		Peak 2 of the acoustic signal (110-2000 hertz).		Signal-to-noise ratio:
		Diagnosis:				Left: 5.7%-11.6% (mean: 16%);
		healthy				Right: 6.1%-13.0% (mean: 16%);
						Acoustic signal peak 1:
						Left: 19.9%-46.6% (mean: 37.3%);
						Right: 22.9%-44.4% (mean: 38.7%);
						Acoustic signal peak 2:
						Left: 26.6%-81.7% (mean: 54.6%);
						Right: 25.1%-81.8% (mean: 54.6%)
Kjellin et al.^(38)^, 1994, Sweden	To elucidate whether the Rapid Oral Suction Swallow (ROSS) test can differentiate between normal and altered swallowing and whether it can strengthen the hypothesis that sequential swallowing (ROSS) is more automatic than single bolus swallowing.	Sample size: 35 people in three groups:	Pressure sensor in the straw and microphone to capture swallowing sounds	Suction duration;	Utensil: straw attached to a cup.	Results presented in graphs without mentioning the exact CV values ​​for each measurement investigated;
		G1 - healthy (9 men and 3 women);		Sub-atmospheric peak suction pressure;	Consistency: liquid (water).	Food bolus transit time: in the ROSS test, the CV of G1 (CV<20%) was significantly lower than that of G2 and G3 (both CV>20%).
		G2 - with complaints of swallowing disorders and normal VFD (10 men and 5 women);		Suction speed;	Volume:	Overall average of the CVs of all measures investigated with the 1st and 2nd tests together: the CV of G1 (CV<20%) was significantly lower than that of G2 and G3 (both CV>20%).
		G3 - with complaints of swallowing disorders and altered VFD (6 men and 2 women).		Food bolus volume;	1st test (single bolus): suck 2x through the straw and swallow the volume calmly;	In general, the CVs followed a pattern: higher for G3, lower for G1, and intermediate for G2.
		Age range:		Food bolus transit time;	2nd test (ROSS): empty the cup as quickly as possible	
		G1: 45 (median); 27-66 (min-max);		Interval time between sucking-swallowing cycles.	Total volume of liquid in the cup: NR.	
		G2: 48 years (median); 37-79 (min-max);				
		G3: 63 years (median); 20-83 (min-max);				
		Diagnosis:				
		Healthy people, gastroesophageal reflux, stroke, CNS tumor, multiple sclerosis, frontal lobe lesion.				
Hughes et al.^(39)^, 1995, United Kingdom	To measure two pharyngeal transit time indices (FW20 and FW50) by electrical impedance tomography.	Sample size: 20 people in 3 groups divided by age.	Electrical impedance tomography	Pharyngeal transit time (FW20, FW50 and mean maximum amplitude)	Utensil: NR	FW20:
		G1: 3 men and 4 women			Consistency: liquid (water) and Dioralyte (liquid drug to measure conductivity);	5ml: male (22.2%); female (25.8%)
		G2: 3 men and 3 women			Volume: 5x each volume for each type of liquid: 5ml, 10ml, and	10ml: male (25.9%); female (34.1%)
		G3: 4 men and 3 women			20 ml (total: 30 swallows per participant)	20ml: male (22.5%); female (28.2%)
						FW50:
		Age range:				5ml: male (32.4%); female (19.9%)
		G1: mean of 29.6 years				10ml: male (18.3%); female (25.8%)
		G2: mean of 49.0 years				20ml: male (17.6%); female (14.5%)
		G3: mean of 59.9 years				Maximum average amplitude:
						5ml: male (25.7%); female (22.4%)
		Diagnosis:				10ml: male (21.5%); female (16.8%)
		healthy				20ml: male (15.9%); female (23.1%)
Hughes et al.^(40)^, 1996, Wales	To compare the repeatability of pharyngeal transit time indices (FW20 and FW50) using electrical impedance tomography with the clinical swallowing capacity test	Sample size: 8 people (6 men and 2 women)	Electrical impedance tomography	Pharyngeal transit time (FW20 and FW50)	Utensil: NR	FW20: 19.0% (9.4%-54.2%);
		Age range: 25 to 61 years			Consistency: liquid (water) and Dioralyte (liquid drug to measure conductivity);	FW50: 15.1% (7.2%-26.8%)
		Diagnosis:			Volume: 10x 20ml of each type of liquid (total: 20 swallows per participant)	
		healthy				
Nilsson et al.^(41)^, 1996, Sweden	To establish normative values ​​and variations in swallowing assessed by the Rapid Oral Suction Swallow (ROSS) test	Sample size: 292 people (177 men and 115 women)	Pressure sensor in the straw; piezoelectric sensor; Doppler probe; thermodetector.	Peak suction pressure;	Utensil: straw attached to a cup.	1st test (single bolus):
		Age range: 18 to 64 years (mean: 38±10 years)		Suction time;	Consistency: liquid (water).	Peak suction pressure: 37%
		Diagnosis:		Bolus volume;	Volume:	Suction time: 52%
		healthy		Oropharyngeal transit time;	1st test (single bolus): suck through the straw and swallow once at the participant's usual volume and speed (2x);	Bolus volume: 33%
				Breathing time;	2nd test (ROSS): swallow the rest of the volume as quickly as possible in repeated ingestion cycles.	Oropharyngeal transit time: 64%
				Feeding interval;	Total volume of liquid in the cup: 200ml.	Time for breathing: 42%
				Ingestion cycle time;		2nd test (ROSS):
				Swallowing capacity.		Suction time: 60
						Bolus volume: 39%
						Oropharyngeal transit time: 48%
						Feeding interval: 95%
						Swallowing cycle time: 51%
						Swallowing capacity: 29%
Jones and Ciucci^(6)^, 2016, USA	Using predictive modeling to determine which quantitative swallowing variables best differentiate individuals with early to mid-stage Parkinson's disease from healthy controls.	Sample size: SG: 26 individuals with Parkinson's disease (13 men and 13 women);	High-resolution manometry	Pressure during swallowing in the velopharynx, mesopharynx, and upper esophageal sphincter regions (CV was calculated in each region considering each sensor from the beginning to the end of swallowing)	Utensil: syringe	Velopharyngeal pressure:
		CG: 26 healthy individuals (13 men and 13 women).			Consistency: diluted liquid barium;	SG: 64%
		Age range:			Volume: 10x of 10 ml	CG: 102%
		SG: 50–88 years (69±16 years)				Mesopharyngeal pressure:
		CG: 49–86 years (mean: 69.8±17 years)				SG: 97%
		Diagnosis:				CG: 160%
		SG: early to mid-stage Parkinson's disease				Upper esophageal sphincter pressure:
		CG: healthy individuals				SG: 63%
						CG: 106%
						Total CV (sum of all regions):
						SG: 466%
						CG: 762%
						The original article presented the CV as the result of the ratio between mean and standard deviation. The % was calculated by the authors of this review.
Balasubramanian et al.^(26)^, 2017, USA	To characterize the intraindividual and interindividual variability and the recording site of pharyngeal peristaltic pressure in healthy individuals.	Sample size:	High-resolution manometry	Peak peristaltic pressure during swallowing at positions 2, 3, 4, 5, 6, 7, and 8 cm above the upper margin of the upper esophageal sphincter identified manometrically.	Utensil: syringe	P2: 12% (dry); 14% (5ml); 12% (10ml);
		32 individuals (20 men and 12 women);		“Pharyngeal integral contractility” (PhCI): mean pressure amplitude x duration of contractions x length of the region of interest	Consistency: dry swallow and water;	P3: 12% (dry); 10% (5ml); 13% (10ml);
		Age range: 21-83 years (mean: 34±16 years)			Volume: 3x dry swallow, 5 ml and 10 ml	P4: 8% (dry); 9% (5ml); 9 (5ml);
		Diagnosis:				P5: 7% (dry); 9% (5ml); 7% (10ml)
		healthy				P6: 6% (dry); 6% (5ml); 7% (10ml)
						P7: 7% (dry); 9% (5ml); 5% (10ml)
						P8: 11% (dry); 11% (5ml); 11% (10ml);
						PhCI: 11% (dry); 17% (5ml); 12% (10ml)
						CV ranged from 1% to 40% among the different anatomical regions evaluated, considering the three tasks tested;
						The CV means were <15% in all anatomical regions evaluated in the three tasks;
						In general, there was more variability in the proximal and distal regions of the UES and less variability in the intermediate regions;
						The CV means of the PhCI were not different between the evaluations in the supine and sitting positions;
						In none of the measures were the CV means different when comparing the group of younger individuals (20 to 35 years; n=20) with the total group (n=32)
Hedström et al.^(42)^, 2017, Sweden	To investigate the variability in the penetration and aspiration scale (PAS) score between two consecutive offers of the same volume and consistency in people with head and neck cancer and dysphagia	Sample size:	Videofluoroscopy	PAS score	Utensil:	Thin liquid:
		38 individuals (26 men and 12 women).			syringe or spoon; cup for 20 ml of thin liquid only.	3ml: 29.2%
		Age range: 44-80 years (mean: 63.7±8.0 years).				5ml: 25.4%
		Diagnosis:			Consistency:	10ml: 16.8%
		head and neck cancer and dysphagia			Thin liquid, mildly thick liquid, and extremely thick liquid.	20ml: 45.8%
					Volume:	Mildly thick liquid:
					2x each supply of:	5ml: 45.8%
					Thin liquid (IDDSI level 0): 3, 5, 10 and 20 ml;	Extremely thick liquid:
					Mildly thick liquid (IDDSI level 2): ​​5 ml	3ml: 75.9%
					Extremely thick liquid (IDDSI level 4): 3 ml	
Jones et al.^(43)^, 2017, USA	To determine whether an artificial neural network classification technique could differentiate patients with early to intermediate stage Parkinson's disease (PD) from healthy controls taking into account videofluoroscopy data combined with manometry.	Sample size: SG: 31 individuals with Parkinson's disease (17 men and 14 women);	High-resolution pharyngeal manometry and simultaneous videofluoroscopy.	Pressure during swallowing in the velopharynx, tongue base, hypopharynx, tongue base with hypopharynx, and upper esophageal sphincter regions (CV was calculated for each sensor in the region of interest. The mean CV was calculated based on all CVs in the region of interest. The total CV was calculated by summing the mean CVs of all regions of interest)	Utensil:	Velopharyngeal pressure:
		CG: 31 healthy individuals (17 men and 14 women).			syringe; straw only for swallowing liquid in a comfortable volume.	SG: 78% (2ml); 101% (10ml); 110% (free sips)
		Age range:			Consistency:	GC: 53% (2ml); 62% (10ml); 57% (free sips)
		SG: 68.7 ± 9.9 years (mean)			diluted liquid barium.	Tongue base pressure:
		CG: 69.6 ± 10.1 (mean)			Volume:	SG: 87% (2ml); 155 (10ml); 97% (free sips)
		Diagnosis:			10x each supply of:	GC: 79% (2ml); 91% (10ml); 86% (free sips)
		SG: early to intermediate stage Parkinson's disease			2 ml, 10 ml, and free sips	Hypopharyngeal pressure:
		CG: healthy				SG: 108% (2ml); 88% (10ml); 128% (free sips)
						GC: 88% (2ml); 190% (10ml); 83% (free sips)
		
						Mesopharyngeal pressure:
						SG: 403% (2ml); 617% (10ml); 111% (free sips)
						GC: 617% (2ml); 173% (10ml); 86% (free sips)
						Upper esophageal sphincter pressure:
						SG: 82% (2ml); 98% (10ml); 215% (free sips)
						GC: 80% (2ml); 95% (10ml); 106% (free sips)
						Total CV (sum of all regions):
						SG: 355% (2ml); 543% (10ml); 550% (free sips)
						GC: 300% (2ml); 366% (10ml); 331% (free sips)
						In all volumes, for all regions, the CV was higher for patients with PD except for the mesopharynx in the 2ml swallow.
						The original article presented the CV as the result of the ratio between mean and standard deviation. The calculation in % was performed by the authors of this review
Park et al.^(31)^, 2021, South Korea	To evaluate the reliability of surface electromyography (SEMG) of the suprahyoid and infrahyoid regions during swallowing	Sample size: 10 healthy individuals (9 men and 1 woman).	Surface electromyography (SEMG)	Onset latency, offset latency, duration, peak amplitude latency, maximum amplitude during swallowing and area under the curve of the rectified electromyographic signal	Utensil:	Suprahyoid region:
		Age range:			NR	Onset latency: 38.0% (dry); 37.4% (2ml); 34.0% (5ml); 35.5% (20ml); 45.7% (total)
		29.50±1.18 years (mean)			Consistency:	Offset latency: 14.7% (dry); 12.9% (2ml); 14.3% (5ml); 12.8% (20ml); 17.5% (total)
		Diagnosis:			dry swallow and water.	Duration: 12.9% (dry); 12.0% (2ml); 12.5% ​​(5ml); 13.7% (20ml); 15.8% (total)
		healthy			Volume:	Peak amplitude latency: 30.1% (dry); 25.1% (2ml); 56.5% (5ml); 36.7% (20ml); 57.6% (total)
					5x each of:	Maximum amplitude during swallowing: 17.5% (dry); 19.0% (2ml); 19.0% (5ml); 17.7% (20ml); 24.9% (total)
					dry swallow, 2 ml, saliva, 2 ml, 5 ml and 20 ml of water.	Area under the curve of the rectified electromyographic signal: 30.3% (dry); 25.9% (2ml); 26.1% (5ml); 26.7% (20ml); 36.9% (total)
					Total: 20 swallows	Infrahyoid region:
						Onset latency: 33.6% (dry); 30.7% (2ml); 25.4% (5ml); 25.7% (20ml); 33.2% (total)
						Offset latency: 15.2% (dry); 13.5% (2ml); 12.5% ​​(5ml); 12.3% (20ml); 16.5% (total)
						Duration: 14.7% (dry); 14.6% (2ml); 14.0% (5ml); 12.5% ​​(20ml); 17.2% (total)
						Peak amplitude latency: 33.2% (dry); 24.2% (2ml); 36.0% (5ml); 34.1% (20ml); 44.0% (total)
						Maximum amplitude during swallowing: 19.4% (dry); 15.2% (2ml); 18.4% (5ml); 14.2% (20ml); 20.9% (total)
						Area under the curve of the rectified electromyographic signal: 27.5% (dry); 25.2% (2ml); 25.3% (5ml); 20.6% (20ml); 30.9% (total)
						CV < 30% in both regions: offset latency, duration and maximum amplitude.
Diaz and Stegemöller^(24)^, 2022, USA	To examine the electromyographic activity of the submental and laryngeal regions during swallowing in people with Parkinson's disease, considering the most affected (MAS) and least affected side (LAS) by the disease	Sample size: 35 individuals (15 men and 20 women).	Surface electromyography (SEMG)	Area under the curve and electromyographic peak.	Utensil:	Submental region:
		Age range:			NR	Area under the curve:
		67.7 ± 7.9 (mean)			Consistency:	Thin fluid: 42% (MAS); 47% (LAS)
		Diagnosis:			Thin liquid (water) and thick liquid (pudding).	Thickened fluid: 54% (MAS); 48% (LAS)
		Parkinson's disease			Volume:	Electromyographic peak:
					3x of:	Thin fluid: 36% (MAS); 39% (LAS)
					10 ml thin liquid	Thickened fluid: 39% (MAS); 45% (LAS)
					10 ml thick liquid	Laryngeal region:
						Area under the curve:
						Thin fluid: 32% (MAS); 44% (LAS)
						Thickened fluid: 39% (MAS); 50% (LAS)
						Electromyographic peak:
						Thin fluid: 21% (MAS); 36% (LAS)
						Thickened fluid: 29% (MAS); 41% (LAS)
						The only significant difference in CV between MAS and LAS was in the electromyographic peak measurement, in the laryngeal region, during the swallowing of thin liquid
Ohmori et al.^(30)^, 2022, Japan	To analyze the difference between the SEMG obtained when instructing participants on the tipper (fast) and dipper (slow) swallowing methods and to investigate the effect of the distinction on the reproducibility of the SEMG	Sample size: 9	Surface electromyography (SEMG)	SEMG duration and waveform amplitude between start and end	Utensil: Syringe	CV in duration with distinction of method
		Participants (8 men and 1 woman)			Consistency: Liquid (water)	In tipper:
		Age range: 45±10 years			Volume:	17.8% to 19.7%
		Diagnosis: healthy			4 x 10	In dipper:
					swallows (4 ml of water) with 5 fast swallows (tipper) and 5 slow (dipper).	20.1% to 22.1%
					Total: 40 swallows	CV in duration without distinction of method
						25.6% to 26%
						CV in amplitude with distinction of method
						In tipper:
						15.7% to 26.2%
						In dipper:
						20.6% to 38.6%
						CV in amplitude without distinction of method
						21.2% to 38.7%
						Lower variability with distinction of swallowing method (tipper and dipper) than without distinction.
						The original article presented the CV as a result of the ratio between mean and standard deviation. The % was calculated by the authors of this review.

Legend: N/I=not informed.

The instrumental examinations most used in the studies to help diagnose oropharyngeal dysphagia were VFSS^(36,42)^, electrical impedance tomography^(39,40)^, high-resolution pharyngeal manometry^(6,26,43)^, and surface electromyography^(24,30,31)^. Most studies approached individuals without swallowing difficulties, although some specific populations were also studied, such as people with Parkinson's disease (PD)^(6,24,43)^ and head and neck cancer^(42)^.

The most investigated parameters were pressure in the pharyngeal region and upper esophageal sphincter^(6,26,43)^, latency and amplitude measures in electromyographic responses^(24,30,31)^, and pharyngeal transit time^(36,39,41)^.

The volumes offered more often were 10 ml^(6,24,26,30,39,42)^, 5 ml^(26,31,37,39,42)^, and 20 ml^(31,39,40,42)^, mostly of water or thin liquids served in a syringe. Some studies used a cup and straw to assess swallowing in a free or comfortable volume for ingestion^(38,41-43)^.

The articles focused on using the CV to verify the intraindividual variability of quantitative measures in repetitions of the same swallowing task and compare intraindividual CVs between different groups, whether by age, clinical condition, volumes, or food consistencies. CV values ​​ranged from low to high variability, with quite heterogeneous and non-standardized instruments, parameters, and collection procedures.

## DISCUSSION

This scoping review aimed to map the available evidence on the variability of quantitative measures obtained in instrumental swallowing examinations in adults. The results indicated that the studies prioritized the investigation of intraindividual variability and that the heterogeneity of the examinations, collection procedures, and quantitative parameters contributed to the high CV amplitude, which also limited the comparison between studies.

The review found studies with different types of instrumental swallowing examinations. Some were authored by researchers in common^(6,39,40,43)^, which may justify, in these cases, the occurrence of similar methods and instruments. High-resolution manometry and surface electromyography were the instruments most used in the selected studies. Although the literature indicates VFSS as the reference instrument for the evaluation of this function^(10-12)^, only two studies that investigated CV used this examination directly^(36,42)^.

Nevertheless, the studies analyzed different populations and parameters, although the consistency of the food bolus offered in the swallowing tasks was decisive for the oscillations in CV values. In any case, the data indicate that the measures obtained through VFSS, considered the reference standard for instrumental evaluation of swallowing, do not have well-established intraindividual or interindividual variability.

The studies included in this review have several differences that make comparison difficult, such as the lack of standardization in the nomenclature of consistencies, the diversified volumes, the different utensils, the number of repetitions, and the different parameters investigated. Another important aspect is the small sample sizes in these studies, commonly fewer than 30 participants, which especially compromises the external validity of the results.

These studies provide no robust information on sample calculation, sampling, or whether the CV was calculated in this process. The CV is considered a relatively reliable indicator of a repeated measure or task, being dependent on the proportional changes in the mean and standard deviation of the sample (heteroscedasticity)^(44)^. Therefore, the lack of more solid information on sample selection in these studies weakens the interpretation of the data and is a limitation to be overcome in future studies that analyze variability through CV calculation.

Most studies were conducted with healthy individuals. Those with an underlying disease were restricted to PD^(6,24,43)^, head and neck cancer^(42)^, or multiple diagnoses^(38)^. Hence, researchers have invested more in understanding the variability of quantitative measures obtained in instrumental swallowing tests in people with preserved swallowing function than in dysphagic individuals.

When comparing these groups, intraindividual variability was generally greater in patients with swallowing disorders^(38)^ or a specific underlying disease^(6)^. It is known that intraindividual or interindividual variability may be influenced by individual factors such as sex, age, and anatomical and functional differences. It must be considered that the dispersion of data may be greater when there is also an adverse clinical condition, due to the body's natural adaptations.

Variability can be an important parameter for monitoring and early detection of dysphagic signs and symptoms. A study using high-resolution manometry that investigated pressure in different anatomical regions during swallowing found that pressure variability in the velopharynx helped to distinguish healthy individuals from those with early to intermediate-stage PD^(6)^. It also found that an approach with swallowing assessment associated with complementary tests and protocols can indicate early changes in PD that are not observed in isolated evaluations.

Another example of a study in individuals with PD used surface electromyography^(24)^. Considering the population with PD and low levels of swallowing impairment, a significant difference was found in the variability of the amplitude of the electromyographic peak in the laryngeal region between the most affected side and the side less affected by the disease when swallowing thin liquids. The authors of the study believe that the smaller variation on the most affected side may be the result of the less force produced in the muscles involved in swallowing.

Most outcomes indicated the influence of the swallowed volume. For instance, the CV was higher in the semisolid swallowing task in most measures investigated in one of the studies that used VFSS^(36)^. The other study with VFSS analyzed the penetration aspiration scale (PAS) scores^(42)^. The data showed high variability for 20 ml of thin liquid, 5 ml of mildly thick liquid, and 3 ml of extremely thick liquid. Its authors attribute this high variability to the complexity required from the swallowing mechanism due to larger volumes and the need for multiple swallows in thicker consistencies.

The variability of parameters must be determined to understand which of them present better homogeneity in the repetitions analyzed, and which one is therefore the most reliable for evaluating and interpreting the performance of the investigated function. An example of this is a study that used surface electromyography, whose parameters with the lowest intraindividual variability among the various measures analyzed were offset latency, duration, and maximum amplitude of electromyographic activity during swallowing^(31)^. Another study revealed that variability helped to identify that a voluntarily modifiable swallowing method with instructions may be the most suitable to apply in surface electromyography in clinical practice^(30)^.

Nonetheless, the high intraindividual dispersion poses a challenge to determining values ​​that represent the expected normal range of the quantitative parameters investigated. An example of this complexity is a study that used high-resolution manometry and found that pharyngeal motility generated pressures with different degrees of variability depending on the anatomical region^(26)^.

The different CV results found in each article reflect the heterogeneity of the methods adopted in the studies. The articles were concerned with using CV to verify intraindividual variability by repeating different swallowing tasks at least twice and comparing intraindividual CVs between different groups, whether by age, clinical condition, volume, or food consistencies.

Some limitations were found during this literature mapping. Few articles were eligible because some researchers investigated variability through measures other than CV or used it to evaluate morphometry, strength, resistance, coughing, or respiratory flow. Although these characteristics are associated with swallowing, they do not necessarily evaluate the individual performing this function. The heterogeneity of the methods restricted the comparison between studies and indicated the need for greater standardization of collection and analysis procedures, including the cutoff point for interpreting CV.

## CONCLUSION

Studies that analyzed the variability of quantitative swallowing parameters obtained through instrumental examinations are heterogeneous and indicated that the dispersion of measures ranges from low to high according to the type of examination, parameter, presence or absence of underlying diseases, and characteristics of the food bolus such as consistency and volume.
