# Supplementary material for: Variability in quantitative outcomes of instrumental swallowing assessments in adults: a scoping review
Source: Codas. 2024 Sep 13;36(5):e20240046. doi: 10.1590/2317-1782/20242024046en (PMC11404840; doi:10.1590/2317-1782/20242024046en)
Supplement: Translation

# Translation: Variabilidade dos desfechos quantitativos nas avaliações instrumentais da deglutição em adultos: uma revisão de escopo

**DOI:** 10.1590/2317-1782/20242024046pt

**Authors:** Jayne de Freitas Bandeira, Desiré Dominique Diniz de Magalhães, Leandro Pernambuco

**Affiliations:** 1 Universidade Federal da Paraíba – UFPB - João Pessoa (PB), Brasil.; 2 Universidade Federal de Pernambuco – UFPE - Recife (PE), Brasil.

**Keywords:** Deglutição, Transtornos de Deglutição, Variação Biológica da População, Avaliação em Saúde, Diagnóstico, Técnicas e Procedimentos Diagnósticos

## Abstract

**Objetivo:**

Mapear as evidências científicas sobre a variabilidade dos parâmetros quantitativos extraídos por exames instrumentais de avaliação da deglutição em adultos, mediante o coeficiente de variação.

**Estratégia de pesquisa:**

Foram seguidos os procedimentos metodológicos recomendados pelo Joanna Briggs Institute e a extensão para revisões de escopo do Preferred Reporting Items for Systematic Reviews and Meta-Analysis (PRISMA-ScR).

**Critérios de seleção:**

A busca foi realizada nas bases de dados Pubmed/Medline, Lilacs, Cochrane Library, Embase, Web of Science, Scopus e CINAHL, assim como no Google Scholar para consultar a literatura cinzenta.

**Análise dos dados:**

Dois revisores cegos e independentes fizeram o rastreamento dos artigos por título e resumo. Posteriormente, os artigos foram lidos na íntegra e selecionados de acordo com os critérios de elegibilidade. Os dados foram extraídos de acordo com um instrumento padronizado.

**Resultados:**

Foram encontrados 363 estudos, sendo 13 elegíveis. A maioria dos estudos teve amostra menor que 30 participantes e foi composta por indivíduos saudáveis. Os exames instrumentais utilizados foram diversos: videofluoroscopia, tomografia de impedância elétrica, sensores laríngeos, manometria de alta resolução e eletromiografia de superfície. Os estudos investigaram principalmente a variabilidade intraindividual e os valores do coeficiente de variação oscilaram entre baixa e alta variabilidade, pois os instrumentos, parâmetros e procedimentos de coleta foram heterogêneos e não padronizados.

**Conclusão:**

A variabilidade intraindividual dos parâmetros quantitativos da deglutição obtidos por meio de exames instrumentais em adultos oscila entre baixa e alta conforme o exame, parâmetro testado, presença ou não de doença de base, consistência e volume do bolo alimentar.

## INTRODUÇÃO

A deglutição é uma função complexa que envolve múltiplas estruturas e regiões neuromusculares^(1,2)^. A avaliação clínica inicial consiste na observação estrutural e funcional, a fim de coletar dados, observar sinais e sintomas e realizar o direcionamento do raciocínio clínico^(3)^. Porém, de forma isolada pode ser ineficiente para detectar alterações não visíveis que caracterizam um distúrbio de deglutição^(4)^ prejudicando a eficácia do tratamento^(5)^.

Dessa forma, o uso de exames instrumentais é importante para investigar estes parâmetros de forma mais precisa^(6)^. Com a observação de imagens resultantes desses exames são analisados detalhes das estruturas e obtidos dados mais quantitativos, os quais vem ganhando destaque diante dos avanços tecnológicos^(7)^ por auxiliar no processo de diagnóstico^(5,8)^.

Na prática clínica, o exame de videofluoroscopia da deglutição permite a visualização em tempo real do transporte do alimento por meio de imagens sequenciais videorradiográficas^(9)^. É considerado o procedimento de referência para avaliação e identificação do risco ou ocorrência de penetração ou aspiração do alimento^(9-12)^ promovendo a identificação de distúrbios da deglutição e analisando os efeitos das estratégias de tratamento^(13-15)^.

Outro exame também utilizado para detecção de alterações na deglutição é a videoendoscopia. Este exame fornece a visualização da hipofaringe e laringe, logo possibilita a observação de resíduos, penetração e aspiração em região laringofaríngea^(15)^. Pode ser realizada tanto na população infantil como em adultos^(16)^ e apresenta viabilidade para análise quantitativa do tempo de duração da fase faríngea^(10)^.

A ultrassonografia (USG), por sua vez, vem sendo utilizada de forma complementar na investigação e monitoramento de parâmetros morfométricos e cinemáticos da fase oral e da fase faríngea da deglutição^(17,18)^. Sua aplicabilidade diante das inovações técnicas auxilia no diagnóstico e tratamento da disfagia possibilitando, por exemplo, avaliações de movimento hiolaríngeo e de língua^(19,20)^. Existem ainda exames como a manometria de alta resolução que pode revelar mudanças em medidas de pressão e tempo em faringe e esôfago durante a deglutição, mesmo que não haja queixas ou sinais clínicos aparentes^(6)^.

Devido à complexidade na extração e análise de medidas quantitativas, a grande maioria dos exames necessitam de treinamento e experiência do avaliador^(21-23)^, visto que se observa a dificuldade na padronização dos métodos, além da presença de particularidades intra e inter-indivíduos que impossibilitam a comparação de estudos. Entretanto, esses exames permitem a investigação dos aspectos relacionados ao desempenho da deglutição por meio de imagens e a possibilidade de quantificar dados^(8)^. Logo, a quantificação viabiliza a comparação e o monitoramento da evolução no indivíduo, fornecendo informações complementares e norteadoras para o processo terapêutico^(24)^.

Na literatura, nota-se que a análise de confiabilidade e a validade de instrumentos para avaliação são de extrema importância para verificar a qualidade da informação para diagnóstico^(25)^. Porém, ressalta-se também a contribuição em analisar a variabilidade dos dados quantitativos extraídos por esses exames. A variabilidade ou dispersão é normalmente investigada por meio de indicadores como o coeficiente de variação (CV), calculado pela razão entre o desvio padrão e a média do conjunto de dados^(26)^.

O CV é uma medida adimensional de variabilidade, portanto, permite comparar dados que possuem unidades distintas e expressar o seu resultado em porcentagem^(27)^. Normalmente, o CV dos sistemas biológicos oscila entre 10-15%^(28)^ e quando o CV está acima de 30% indica que a medida possui alta heterogeneidade^(29)^. Dessa forma, quanto menor o CV, menor será o grau de variabilidade^(30)^.

A dispersão pode ser influenciada por vários fatores, tanto biológicos como resultantes dos padrões de avaliação, visto que nas avaliações instrumentais da deglutição pode haver divergências no intervalo de tempo entre as ofertas, consistência do alimento, volume e sequência de tentativas, por exemplo^(31)^. Ao analisar a variabilidade das medidas pode-se compreender a homogeneidade e representatividade dessas, o que ajuda identificar inconsistências^(32)^. A partir disso, pode-se analisar se essas medidas realmente auxiliam a caracterizar o parâmetro de interesse e assim decidir se elas são seguras para serem aplicadas na prática.

Portanto, o objetivo da presente revisão foi mapear as evidências científicas sobre a variabilidade dos parâmetros quantitativos extraídos por exames instrumentais da deglutição, mediante a investigação do CV. Diante disso, o estudo foi norteado pela seguinte pergunta de pesquisa: “Qual o nível de variabilidade dos parâmetros quantitativos de avaliações instrumentais da deglutição em adultos?”.

## MÉTODO

Esta revisão de escopo foi realizada conforme as recomendações metodológicas do *Joanna Briggs Institute (JBI)* para o tipo de estudo em questão^(33)^ e seguiu os critérios do *Preferred Reporting Items for Systematic Reviews and Meta-Analysis* – extensão para revisões de escopo (PRISMA-ScR)^(34)^. O protocolo desta revisão foi publicado previamente e relatou os objetivos, critérios de elegibilidade, fontes e estratégia de busca, seleção, análise e métodos de apresentação dos dados^(35)^. O protocolo deste estudo foi registrado no *Open Science Framework* em 28 de janeiro de 2023 (https://osf.io/p3g2e/). Para definição da pergunta de pesquisa foi considerado o acrônimo PCC formado por: População (indivíduos adultos jovens e/ou idosos que tenham sido submetidos à avaliação da deglutição), Conceito (nível de variabilidade dos dados quantitativos resultantes da avaliação de deglutição) e Contexto (estudos utilizando exames instrumentais que possuam resultados quantitativos do coeficiente de variação para a análise dos parâmetros da deglutição).

### Estratégias de busca

O levantamento bibliográfico foi realizado em 03 de dezembro de 2022 e uma busca atualizada foi realizada em 30 de novembro de 2023 nas bases de dados eletrônicas: *Pubmed/Medline, Lilacs via BVS, Cochrane Library, Embase, Web of Science, Scopus* e *CINAHL via EBSCO*, assim como na literatura cinzenta do *Google Scholar*, sendo neste último considerado apenas os 100 primeiros artigos da busca. Não foi possível realizar a busca na base de dados *Proquest* conforme previsto no protocolo^(35)^, visto que o acesso não estava liberado para a instituição dos autores no momento da busca.

A estratégia de busca foi realizada a partir da combinação de descritores e palavras-chave (Quadro 1) adaptadas para cada base de dados. Também foram consideradas as referências contidas nos artigos encontrados e verificadas de forma manual a fim de identificar estudos que demonstrassem relevância com o tema de interesse.

**Quadro 1:** Estratégias de busca nas bases de dados

**Base de dados**	**Descritores**	**Registros Encontrados 03.12.22**	**Registros Encontrados 30.11.23**
**PubMed/Medline**	(((((((((deglutition disorders[MeSH Terms]) OR (dysphagia[MeSH Terms])) OR (swallowing disorders[MeSH Terms])) OR (deglutition[MeSH Terms])) OR (swallowing[MeSH Terms])) OR (dysphagia)) OR (deglutit*)) OR (swallow*)) AND (“coefficient of variation” [All Fields]))	56	57
**Lilacs via BVS**	(“deglutition disorders” OR “dysphagia” OR “swallowing disorder” OR swallow* OR deglutit*) AND (“coefficient of variation”)	4	4
**Cochrane Library**	Dysphagia AND coefficient of variation	3	4
**Embase**	('swallowing'/exp OR swallowing OR 'dysphagia'/exp OR dysphagia) AND ('coefficient of variation'/exp OR 'coefficient of variation')	63	64
**Web of Science**	(TS=((“deglutition” OR “deglutitions” OR “swallowing” OR “swallowings” OR “swallow” OR “swallows” OR “deglutition disorders” OR “deglutition disorder” OR “swallowing disorder” OR “swallowing disorders” OR “dysphagia”) AND (“coefficient of variation”)))	46	46
**SCOPUS**	(TITLE-ABS-KEY((“Dysphagia” OR “Swallowing disorder” OR “Deglutition” OR”Deglutition Disorders”OR “Swallowing”)) AND TITLE-ABS-KEY((“coefficient of variation”)))	61	62
**CINAHL via EBSCO**	Dysphagia OR Swallowing disorders OR Deglutition OR Swallowing OR Deglutition Disorders AND coefficient of variation	30 Texto completo	40 Texto completo
**Google Scholar**	(“Dysphagia” OR “Swallowing disorders”) AND (“coefficient of variation”)	2160 Considerado apenas os 100 primeiros estudos.	2440 Considerado apenas os 100 primeiros estudos.
**TOTAL**		363	377

### Seleção dos estudos

Após a busca foram seguidas as seguintes etapas:

1. Os artigos recrutados em cada base foram importados para o *software EndNote (Clarivate Analytics, PA, EUA)* para o gerenciamento e remoção dos estudos duplicados;
2. Utilizou-se o *software Rayyan (Qatar Computing Research Institute, Doha, Qatar)* para triagem dos artigos pelo título e resumo de modo cego entre os dois revisores;
3. Os conflitos foram resolvidos a partir da análise de um terceiro revisor.

Foram incluídos todos os artigos disponíveis na íntegra, sem restrição de ano e de idioma, nas fontes de evidência estabelecidas. Foram excluídos estudos que não analisaram o nível de variabilidade por meio do CV, que não informaram a média e o desvio padrão para possibilitar o cálculo do CV pelos pesquisadores e os que utilizaram fórmulas alternativas para o cálculo do CV. Além disso, foram excluídos estudos que utilizaram exames instrumentais, mas não apresentaram resultados quantitativos, estudos que avaliaram apenas a deglutição esofágica e aqueles desenvolvidos apenas com a população infantil.

### Extração e apresentação dos dados

Os estudos incluídos foram selecionados a partir das etapas apresentadas no diagrama de fluxo preconizado pelo *PRISMA-ScR* ^(34)^. Após os filtros iniciais, os artigos incluídos foram analisados por meio da leitura do texto completo pela dupla de revisores e considerados os critérios de elegibilidade para sua manutenção no resultado final. Os dados foram extraídos de acordo com os objetivos da pesquisa seguindo uma matriz de extração previamente apresentada no protocolo desta revisão^(35)^.

## RESULTADOS

Após o processo de busca e seleção dos 363 estudos encontrados, 13 foram elegíveis^(6,24,26,30,31,36-43)^ pelos critérios de inclusão estabelecidos (Figura 1).

**Figura 1:** Fluxograma do processo de seleção dos estudos - PRISMA-ScR (2020)

Os dados extraídos dos artigos incluídos nesta revisão estão no Quadro 2. Os estudos foram publicados entre os anos de 1990 e 2022, sendo seis deles^(6,24,26,36,37,43)^ desenvolvidos nos Estados Unidos. Em relação à amostra, seis (46,15%) estudos^(30,31,36,37,39,40)^ apresentaram uma amostra menor que 30 participantes. Mais de 50% das pesquisas foram realizadas com uma população mista quanto ao gênero (homens e mulheres) e compostas por indivíduos adultos jovens e idosos. Dois artigos (15,3%) mencionaram o pareamento da amostra por sexo^(6,43)^.

**Quadro 2:** Matriz de análise dos estudos que utilizaram o CV para investigar a variabilidade dos parâmetros ou desfechos quantitativos extraídos por exames instrumentais da deglutição

**Autor, Ano, País**	**Objetivo do estudo**	**Tamanho da amostra, Faixa etária, Diagnóstico**	**Exame**	**Parâmetros investigados**	**Utensílio, Volume, Consistência de oferta**	**Coeficiente de variação (CV)**
Lof e Robbins^(36)^, 1990, EUA	Determinar se os parâmetros temporais da deglutição em sujeitos saudáveis permanecem estáveis no teste-reteste.	Tamanho da amostra: 8 mulheres e 8 homens	Videofluoroscopia	Duração do trânsito oral (OTD); Duração do estágio de transição (STD);	Utensílio: colher	*OTD* = 50% (Lq), 43% (Ss);
				Duração do trânsito faríngeo (PTD);	Consistência de oferta: bário líquido (Lq) e semi-sólido (Ss)	*STD* = 1.14% (Lq), 17.67% (Ss);
		Faixa etária:		Duração da resposta faríngea (PRD);	Volume: 3x 2ml de cada consistência	*PRD* = 21% (Lq), 36% (Ss);
		grupo 1: 43-45 anos		Duração da excursão do véu palatino (DOVE);		*PTD* = 18% (Lq), 59% (Ss);
		grupo 2: 63-67 anos		Duração da máxima elevação do hioide (DOHME);		*DOVE* =28% (Lq), 41% (Ss);
		Diagnóstico: saudáveis		Duração da máxima anteriorização do hioide (DOHMA);		*DOHME* = 48% (Lq), 63% (Ss);
				Duração da abertura do esfíncter esofágico superior (DOOUES);		*DOHMA* = 45 (Lq), 38% (Ss);
				Duração até a abertura do esfíncter esofágico superior (DTOUES).		*DTOUES* = 38% (Lq), 38% (Ss);
						*DOOUES* = 50% (Lq), 43% (Ss).
Takahashi et al.^(37)^, 1994, EUA	Investigar a simetria e reprodutibilidade dos sons da deglutição detectados bilateralmente em indivíduos saudáveis.	Tamanho da amostra: 5 mulheres e 5 homens	Sonógrafo e acelerômetros	Duração dos sons (em segundos);	Utensílio: N/I	*Duração dos sons:*
		Faixa etária:		Relação sinal-ruído (em decibéis);	Consistência de oferta: líquido (água)	Esquerda: 18.7%-61.4% (média: 34.0%);
		homens - 35.4 anos (média)		Pico 1 do sinal acústico (<110 hertz);	Volume: 30x 5ml	Direita: 17.9%-66.3% (média: 35.3%);
		mulheres - 29.4 anos (média)		Pico 2 do sinal acústico (110-2000 hertz).		*Relação sinal-ruído:*
		Diagnóstico:				Esquerda: 5.7%-11.6% (média: 16%);
		saudáveis				Direita: 6.1%-13.0% (média: 16%);
						*Pico 1 do sinal acústico:*
						Esquerda: 19.9%-46.6% (média: 37.3%);
						Direita: 22.9%-44.4% (média: 38.7%);
						*Pico 2 do sinal acústico:*
						Esquerda: 26.6%-81.7% (média: 54.6%);
						Direita: 25.1%-81.8% (média: 54.6%);
Kjellin et al.^(38)^, 1994, Suécia	Elucidar se o teste *Rapid*	Tamanho da amostra: 35 pessoas em três grupos:	Sensor de pressão no canudo e microfone para captação dos sons da deglutição	Duração da sucção;	Utensílio: canudo fixado em copo.	Resultados apresentados em gráficos sem menção aos valores exatos do CV em cada medida investigada;
	*Oral Suction Swallow (ROSS)* pode diferenciar a deglutição entre normal e alterada e se pode fortalecer a hipótese de que a deglutição sequencial (ROSS) é mais automática	G1 - saudáveis (9 homens e 3 mulheres);		Pico subatmosférico da pressão de sucção;	Consistência de oferta: líquido (água).	*Tempo de trânsito do bolo alimentar*: no teste ROSS, o CV do G1 (CV<20%) foi significativamente menor que G2 e G3 (ambos CV>20%).
	do que a deglutição em bolo único.	G2 - com queixa de alteração na deglutição e VFD normal (10 homens e 5 mulheres);		Velocidade de sucção;	Volume:	*Média geral dos CV de todas as medidas investigadas com o 1º e 2º testes juntos*: o CV do G1 (CV<20%) foi significativamente menor que G2 e G3 (ambos CV>20%).
		G3 - com queixa de alteração na deglutição e VFD alterada (6 homens e 2 mulheres).		Volume do bolo alimentar;	1º teste (bolo único): 2x sugar pelo canudo e deglutir o volume calmamente;	De forma geral, os CV seguiram um padrão: maiores para G3, menores para G1 e intermediários para G2.
				Tempo de trânsito do bolo alimentar;	2º teste (ROSS): esvaziar o copo o mais rápido possível	
		Faixa etária:		Tempo de intervalo entre os ciclos de sucção-deglutição.	Volume total de líquido no copo: N/I.	
		G1: 45 (mediana); 27-66 (mín-máx);				
		G2: 48 anos (mediana); 37-79 (mín-máx);				
		G3: 63 anos (mediana); 20-83 (mín-máx);				
		Diagnóstico:				
		Pessoas saudáveis, refluxo gastroesofágico, AVC, tumor de SNC, esclerose múltipla, lesão de lobo frontal.				
Hughes et al.^(39)^, 1995, Reino Unido	Medir dois índices do tempo de trânsito faríngeo (FW20 e FW50) por meio de tomografia de impedância elétrica.	Tamanho da amostra: 20 pessoas em 3 grupos divididos por idade.	Tomografia de impedância elétrica	Tempo de trânsito faríngeo (FW20, FW50 e amplitude média máxima).	Utensílio: N/I	*FW20:*
		G1: 3 homens e 4 mulheres			Consistê ncia de oferta: líquido (água) e *Dioralyte* (fármaco líquido para mensurar condutividade);	5ml: homem (22.2%); mulher (25.8%)
		G2: 3 homens e 3 mulheres			Volume: 5x cada volume para cada tipo de líquido: 5ml, 10ml e	10ml: homem (25.9%); mulher (34.1%)
		G3: 4 homens e 3 mulheres			20 ml (total: 30 deglutições por participante)	20ml: homem (22.5%); mulher (28.2%)
		Faixa etária:				*FW50:*
		G1: média de 29,6 anos				5ml: homem (32.4%); mulher (19.9%)
		G2: média de 49,0 anos				10ml: homem (18.3%); mulher (25.8%)
		G3: média de 59,9 anos				20ml: homem (17.6%); mulher (14.5%)
						*Amplitude média máxima:*
		Diagnóstico:				5ml: homem (25.7%); mulher (22.4%)
		saudáveis				10ml: homem (21.5%); mulher (16.8%)
						20ml: homem (15.9%); mulher (23.1%)
Hughes et al.^(40)^, 1996, País de Gales	Comparar a repetibilidade de índices do tempo de trânsito faríngeo (FW20 e FW50) por meio de tomografia de impedância elétrica com o teste clínico de capacidade de deglutição.	Tamanho da amostra: 8 pessoas (6 homens e 2 mulheres)	Tomografia de impedância elétrica	Tempo de trânsito faríngeo (FW20 e FW50).	Utensílio: N/I	*FW20*: 19.0% (9.4%-54.2%);
		Faixa etária: 25 a 61 anos			Consistê ncia de oferta: líquido (água) e *Dioralyte* (fármaco líquido para mensurar condutividade);	*FW50*: 15.1% (7.2%-26.8%)
		Diagnóstico:			Volume: 10x de 20ml de cada tipo de líquido (total: 20 deglutições por participante)	
		saudáveis				
Nilsson et al.^(41)^, 1996, Suécia	Estabelecer valores normativos e de variações da deglutição avaliada pelo teste *Rapid*	Tamanho da amostra: 292 pessoas (177 homens e 115 mulheres)	Sensor de pressão no canudo; sensor piezo elétrico; sonda Doppler; termodetector.	Pico de pressão de sucção;	Utensílio: canudo fixado em copo.	*1º teste (bolo único):*
	*Oral Suction Swallow (ROSS).*	Faixa etária: 18 a 64 anos (média: 38±10 anos)		Tempo de sucção;	Consistência de oferta: líquido (água).	Pico de pressão de sucção: 37%
		Diagnóstico:		Volume do bolo;	Volume:	Tempo de sucção: 52%
		saudáveis		Tempo de trânsito orofaríngeo;	1º teste (bolo único): sugar pelo canudo e deglutir uma única vez em volume e velocidade usuais para o participante (2x);	Volume do bolo: 33%
				Tempo para respiração;	2º teste (ROSS): deglutir o resto do volume o mais rápido possível em repetidos ciclos de ingestão.	Tempo de trânsito orofaríngeo: 64%
				Intervalo de alimentação;	Volume total de líquido no copo: 200ml.	Tempo para respiração: 42%
				Tempo do ciclo de ingestão;		*2º teste (ROSS):*
				Capacidade de deglutição.		Tempo de sucção: 60
						Volume do bolo: 39%
						Tempo de trânsito orofaríngeo: 48%
						Intervalo de alimentação: 95%
						Tempo do ciclo de ingestão: 51%
						Capacidade de deglutição: 29%
Jones e Ciucci^(6)^, 2016, EUA	Usar modelagem preditiva para determinar quais variáveis quantitativas de deglutição diferenciam melhor indivíduos com Doença de Parkinson em estágio inicial a intermediário de controles saudáveis.	Tamanho da amostra: GE: 26 indivíduos com doença de Parkinson (13 homens e 13 mulheres);	Manometria de alta resolução	Pressão durante a deglutição nas regiões de velofaringe, mesofaringe, esfíncter esofágico superior (CV foi calculado em cada região considerando cada sensor desde o início até o final da deglutição).	Utensílio: seringa	*Pressão na velofaringe:*
		GC: 26 indivíduos saudáveis (13 homens e 13 mulheres).			Consistência de oferta: bário líquido diluído;	GE: 64%
		Faixa etária:			Volume: 10x de 10ml	GC: 102%
		GE: 50-88 anos (69±16 anos)				*Pressão na mesofaringe:*
		GC: 49–86 anos (média: 69.8±17 anos)				GE: 97%
		Diagnóstico:				GC: 160%
		GE: doença de Parkinson em estágio inicial a intermediário				*Pressão no esfíncter esofágico superior:*
		GC: saudáveis				GE: 63%
						GC: 106%
						*Total do CV (soma de todas as regiões):*
						GE: 466%
						GC: 762%
						*O artigo original apresentou o CV como resultado da razão entre média e desvio padrão. O cálculo em % foi realizado pelos autores desta revisão.*
Balasubramanian et al.^(26)^, 2017, EUA	Caracterizar em indivíduos saudáveis a variabilidade intra e interindividual e o local de registro da pressão peristáltica faríngea.	Tamanho da amostra:	Manometria de alta resolução	Pico da pressão peristáltica durante a deglutição nas posições 2, 3, 4, 5, 6, 7 e 8 cm acima da margem superior do esfíncter esofágico superior identificado manometricamente.	Utensílio: seringa	*P2*:12% (seca); 14% (5ml); 12% (10ml);
		32 indivíduos (20 homens e 12 mulheres);		“Contratilidade integral da faringe” (PhCI): média da amplitude da pressão x duração das contrações x comprimento da região de interesse.	Consistê ncia de oferta: deglutição seca e água;	*P3*:12% (seca); 10% (5ml); 13% (10ml);
		Faixa etária: 21-83 anos (média: 34±16 anos)			Volume: 3x deglutição seca, 5ml e 10ml	*P4*: 8% (seca); 9% (5ml); 9 (5ml);
		Diagnóstico:				*P5*: 7% (seca); 9% (5ml); 7% (10ml)
		saudáveis				*P6*: 6% (seca); 6% (5ml); 7% (10ml)
						*P7*: 7% (seca); 9% (5ml); 5% (10ml)
						*P8*:11% (seca); 11% (5ml); 11% (10ml);
						*PhCI*:11% (seca); 17% (5ml); 12% (10ml)
						CV variou de 1% a 40% entre as diferentes regiões anatômicas avaliadas, considerando as três tarefas testadas;
						As médias do CV ficaram <15% em todas as regiões anatômicas avaliadas nas três tarefas testadas;
						De modo geral, houve mais variabilidade nas regiões proximais e distais do EES e menos variabilidade nas regiões intermediárias;
						Médias do CV do PhCI não foram diferentes entre as avaliações nas posições supino e sentado;
						Em nenhuma das medidas as médias do CV foram diferentes ao comparar o grupo de indivíduos mais jovens (20 a 35 anos; n=20) com o grupo total (n=32).
Hedström et al.^(42)^, 2017, Suécia	Investigar a variabilidade no escore da escala de aspiração e penetração (PAS) entre duas ofertas consecutivas de mesmo volume e consistência em pessoas com câncer de cabeça e pescoço e disfagia.	Tamanho da amostra:	Videofluoroscopia	Escore da escala PAS.	Utensílio:	*Líquido fino:*
		38 indivíduos (26 homens e 12 mulheres).			seringa ou colher; copo apenas para 20 ml de líquido fino.	3ml: 29,2%
		Faixa etária: 44-80 anos (média: 63.7±8.0 anos).			Consistência de oferta:	5ml: 25,4%
		Diagnóstico:			Líquido fino, líquido moderadamente espessado e líquido extremamente espessado.	10ml: 16,8%
		câncer de cabeça e pescoço e disfagia.			Volume:	20ml: 45,8%
					2x cada oferta de:	
					Líquido fino (IDDSI nível 0): 3, 5, 10 e 20 ml;	*Líquido levemente espessado:*
					Líquido levemente espessado (IDDSI nível 2): 5 ml	5ml: 45,8%
					Líquido extremamente espessado (IDDSI nível 4): 3 ml	*Líquido extremamente espessado:*
						3ml: 75,9%
Jones et al.^(43)^, 2017, EUA	Determinar se a técnica de classificação de rede neural artificial poderia diferenciar os pacientes com Doença de Parkinson (DP) em estágio inicial a intermediário e controles saudáveis levando em consideração os dados da videofluoroscopia combinados com a manometria.	Tamanho da amostra: GE: 31 indivíduos com doença de Parkinson (17 homens e 14 mulheres);	Manometria de alta resolução da faringe e videofluoroscopia simultânea.	Pressão durante a deglutição nas regiões de velofaringe, base de língua, hipofaringe, base de língua com hipofaringe e esfíncter esofágico superior (CV foi calculado para cada sensor na região de interesse. A média do CV foi calculada com base em todos os CV da região de interesse. O CV total foi calculado pela soma das médias dos CV de todas as regiões de interesse).	Utensílio:	*Pressão na velofaringe:*
		GC: 31 indivíduos saudáveis (17 homens e 14 mulheres).			seringa; canudo apenas para deglutição do líquido em volume confortável.	GE: 78% (2ml); 101% (10ml); 110% (goles livres)
		Faixa etária:			Consistência de oferta:	GC: 53% (2ml); 62% (10ml); 57% (goles livres)
		GE: 68.7 ± 9.9 anos (média)			bário líquido diluído.	*Pressão na base língua:*
		GC: 69.6 ± 10.1 (média)			Volume:	GE: 87% (2ml); 155 (10ml); 97% (goles livres)
		Diagnóstico:			10x cada oferta de:	GC: 79% (2ml); 91% (10ml); 86% (goles livres)
		GE: doença de Parkinson em estágio inicial a intermediário			2 ml, 10 ml e goles livres.	*Pressão hipofaringe:*
		GC: saudáveis				GE: 108% (2ml); 88% (10ml); 128% (goles livres)
						GC: 88% (2ml); 190% (10ml); 83% (goles livres)
						*Pressão mesofaringe:*
						GE: 403% (2ml); 617% (10ml); 111% (goles livres)
						GC: 617% (2ml); 173% (10ml); 86% (goles livres)
						*Pressão no esfíncter esofágico superior:*
						GE: 82% (2ml); 98% (10ml); 215% (goles livres)
						GC: 80% (2ml); 95% (10ml); 106% (goles livres)
						*Total do CV (soma de todas as regiões):*
						GE: 355% (2ml); 543% (10ml); 550% (goles livres)
						GC: 300% (2ml); 366% (10ml); 331% (goles livres)
						Em todos volumes, para todas as regiões, o CV foi maior para os pacientes com DP com exceção da mesofarínge na deglutição de 2ml.
						*O artigo original apresentou o CV como resultado da razão entre média e desvio padrão. O cálculo em % foi realizado pelos autores desta revisão.*
Park et al.^(31)^, 2021, Coréia do Sul	Avaliar a confiabilidade da eletromiografia de superfície (EMGS) das regiões supra e infrahioidea durante a deglutição.	Tamanho da amostra: 10 indivíduos saudáveis (09 homens e 01 mulher).	Eletromiografia de superfície (EMGS)	Latência do *onset*, latência do *offset*, duração, latência do pico de amplitude, amplitude máxima durante a deglutição e área sob a curva do sinal eletromiográfico retificado.	Utensílio:	*Região suprahioidea,:*
		Faixa etária:			N/I	*Latência do onset:* 38.0% (seca); 37.4% (2ml); 34.0% (5ml); 35.5% (20ml); 45.7% (total)
		29,50±1,18 anos (média)			Consistência de oferta:	*Latência do offset:*14.7% (seca); 12.9% (2ml); 14.3% (5ml); 12.8% (20ml); 17.5% (total)
		Diagnóstico:			deglutição seca e água.	*Duração*: 12.9% (seca); 12.0% (2ml); 12.5% (5ml); 13.7% (20ml); 15.8% (total)
		saudáveis			Volume:	*Latência do pico de amplitude*: 30.1% (seca); 25.1% (2ml); 56.5% (5ml); 36.7% (20ml); 57.6% (total)
					5x cada de:	*Amplitude máxima durante a deglutição*: 17.5% (seca); 19.0% (2ml); 19.0% (5ml); 17.7% (20ml); 24.9% (total)
					deglutição seca, 2 ml, saliva, 2 ml, 5 ml e 20 ml de água.	*Área sob a curva do sinal eletromiográfico retificado*: 30.3% (seca); 25.9% (2ml); 26.1% (5ml); 26.7% (20ml); 36.9% (total)
					Total: 20 deglutições.	*Região infrahioidea:*
						*Latência do onset:* 33,6% (seca); 30.7% (2ml); 25.4% (5ml); 25.7% (20ml); 33.2% (total)
						*Latência do offset:*15.2% (seca); 13.5% (2ml); 12.5% (5ml); 12.3% (20ml); 16.5% (total)
						*Duração*: 14.7% (seca); 14.6% (2ml); 14.0% (5ml); 12.5% (20ml); 17.2% (total)
						*Latência do pico de amplitude*: 33.2% (seca); 24.2% (2ml); 36.0% (5ml); 34.1% (20ml); 44.0% (total)
						*Amplitude máxima durante a deglutição*: 19.4% (seca); 15.2% (2ml); 18.4% (5ml); 14.2% (20ml); 20.9% (total)
						*Área sob a curva do sinal eletromiográfico retificado*: 27.5% (seca); 25.2% (2ml); 25.3% (5ml); 20.6% (20ml); 30.9% (total)
						CV < 30% em ambas as regiões: latência do offset, duração e amplitude máxima.
Diaz e Stegemöller^(24)^, 2022, EUA	Examinar a atividade eletromiográfica das regiões submentoniana e laríngea durante a deglutição em	Tamanho da amostra: 35 indivíduos (15 homens e 20 mulheres).	Eletromiografia de superfície (EMGS)	Área sob a curva e pico eletromiográfico.	Utensílio:	*Região submentoniana:*
	pessoas com doença de Parkinson (DP), considerando o lado mais afetado (MAS) e	Faixa etária:			N/I	*Área sob a curva:*
	o lado menos afetado (LAS) pela doença.	67,7 ± 7,9 (média)			Consistência de oferta:	Líquido fino: 42% (MAS); 47% (LAS)
		Diagnóstico:			Líquido fino (água) e espessado (pudim).	Líquido espessado: 54% (MAS); 48% (LAS)
		Doença de Parkinson			Volume:	*Pico eletromiográfico:*
					3x de:	Líquido fino: 36% (MAS); 39% (LAS)
					10 ml de líquido fino	Líquido espessado: 39% (MAS); 45% (LAS)
					10 ml líquido espessado	
						*Região laríngea:*
						*Área sob a curva:*
						Líquido fino: 32% (MAS); 44% (LAS)
						Líquido espessado: 39% (MAS); 50% (LAS)
						*Pico eletromiográfico:*
						Líquido fino: 21% (MAS); 36% (LAS)
						Líquido espessado: 29% (MAS); 41% (LAS)
						A única diferença significativa dos CV entre MAS e LAS foi na medida pico eletromiográfico, na região laríngea, durante a deglutição de líquido fino.
Ohmori et al.^(30)^, 2022, Japão	Analisar a diferença entre os EMGs	Tamanho da amostra: 9	Eletromiografia de Superfície (EMGS).	Duração do EMGS e a amplitude da forma de onda entre o início e o fim.	Utensílio: Seringa	*CV na duração com distinção do método*
	obtidos ao instruir os participantes sobre os métodos de deglutição *tipper* (rápida) e *dipper* (lenta) e investigar o efeito da distinção na reprodutibilidade dos EMGs.	Participantes (8 homens e 1 mulher)			Consistência de oferta: Líquido (água)	No tipper:
		Faixa etária: 45±10 anos				17,8% a 19,7%
		Diagnóstico: saudáveis			Volume:	No dipper:
					4 x de 10	20,1% a 22,1%
					deglutições (4 ml de água) sendo 5 deglutições rápidas (tipper) e 5 lentas (dipper).	
					Total: 40 deglutições.	*CV na duração sem distinção do método*
						25,6% a 26%
						*CV na amplitude com distinção do método*
						No tipper:
						15,7% a 26,2%
						No dipper:
						20,6% a 38,6%
						*CV na amplitude sem distinção do método*
						21,2% a 38,7%
						Variabilidade menor com a distinção do método de deglutição (tipper e dipper) do que sem a distinção.
						*O artigo original apresentou o CV como resultado da razão entre média e desvio padrão. O cálculo em % foi realizado pelos autores desta revisão.*

N/I: não informado.

Observou-se que nos estudos incluídos, os exames instrumentais mais utilizados para auxiliar no diagnóstico de disfagia orofaríngea foram a videofluoroscopia da deglutição^(36,42)^, tomografia de impedância elétrica^(39,40)^, manometria faríngea de alta resolução^(6,26,43)^ e eletromiografia de superfície^(24,30,31)^. Dentre os grupos de participantes, os estudos com indivíduos sem dificuldades na deglutição foram a maioria, porém algumas populações específicas também foram estudadas como pessoas com Doença de Parkinson^(6,24,43)^ e câncer de cabeça e pescoço^(42)^.

Os parâmetros mais investigados foram a pressão em região faríngea e de esfíncter esofágico superior^(6,26,43)^, as medidas de latência e amplitude nas respostas eletromiográficas^(24,30,31)^ e o tempo de trânsito faríngeo^(36,39,41)^.

Os volumes de oferta mais observados foram 10 ml^(6,24,26,30,39,42)^, 5 ml^(26,31,37,39,42)^ e 20 ml^(31,39,40,42)^, sendo administrado, em grande maioria, água ou líquido na consistência fina, utilizando seringa. Em alguns estudos foi utilizado o copo com canudo devido à avaliação da deglutição em volume livre ou confortável para ingestão^(38,41-43)^.

Os artigos se preocuparam em usar o CV para verificar a variabilidade intraindividual de medidas quantitativas nas repetições de uma mesma tarefa de deglutição e comparar os CV intraindividuais entre grupos distintos, seja por idade, condição clínica, volumes ou consistências alimentares. Os valores de CV oscilaram entre baixa e alta variabilidade, com instrumentos, parâmetros e procedimentos de coleta muito heterogêneos e não padronizados.

## DISCUSSÃO

Esta revisão de escopo teve como objetivo mapear a evidência disponível sobre a variabilidade de medidas quantitativas obtidas em exames instrumentais de avaliação da deglutição em adultos. Os resultados indicaram que os estudos priorizaram investigar a variabilidade intraindividual e que a heterogeneidade dos exames, procedimentos de coleta e parâmetros quantitativos contribuiu para a elevada amplitude dos coeficientes de variação, o que inclusive limitou as possibilidades de comparações entre os estudos.

Foram encontradas pesquisas com diferentes tipos de exames instrumentais para avaliação da deglutição. Alguns tinham autoria de pesquisadores em comum^(6,39,40,43)^ podendo justificar, nesses casos, a ocorrência de métodos e instrumentos semelhantes. A manometria de alta resolução e a eletromiografia de superfície foram os instrumentos mais encontrados nos estudos selecionados. Apesar de a literatura apontar a videofluoroscopia da deglutição (VFD) como instrumento de referência na avaliação da função^(10-12)^, apenas dois estudos que investigaram o CV utilizaram esse exame diretamente^(36,42)^.

Ainda assim, as populações e os parâmetros analisados foram diferentes nesses estudos, embora em ambos a consistência do bolo alimentar oferecido nas tarefas de deglutição tenha sido determinante para as oscilações nos valores do CV. De todo modo, os dados apontam que as medidas obtidas por meio da videofluoroscopia, considerado padrão de referência para avaliação instrumental da deglutição, não possuem a variabilidade intra e interindividual bem estabelecidas.

Os estudos incluídos nesta revisão apresentam entre eles várias divergências que dificultam a comparação, como a falta de padronização na nomenclatura das consistências, a diversificação nos volumes utilizados, os diferentes utensílios utilizados, o número de repetições solicitadas e os distintos parâmetros investigados. Outro aspecto importante diz respeito aos pequenos tamanhos de amostra encontrados nessas pesquisas, comumente menos de 30 participantes, o que compromete especialmente a validade externa dos resultados.

Nos estudos incluídos, não há informações robustas sobre o cálculo amostral e amostragem, tampouco se o cálculo do CV foi considerado nesse processo. O CV é considerado um indicador de confiabilidade relativa de uma medida ou tarefa repetida, sendo dependente das mudanças proporcionais da média e desvio padrão da amostra (heterocedasticidade)^(44)^, portanto, a ausência de informações mais sólidas sobre a seleção da amostra nos estudos incluídos fragiliza a interpretação dos dados encontrados e deve ser uma limitação a ser superada em próximos estudos que adotarem a análise da variabilidade por meio do cálculo do CV.

A maioria das pesquisas foi realizada com indivíduos saudáveis e naqueles casos em que havia doença de base, essas foram restritas à doença de Parkinson^(6,24,43)^, câncer de cabeça e pescoço^(42)^ ou múltiplos diagnósticos^(38)^. Há, portanto, um investimento maior dos pesquisadores em compreender a variabilidade das medidas quantitativas obtidas em exames instrumentais da deglutição em pessoas com função de deglutição preservada quando comparadas aos indivíduos disfágicos.

Ao comparar esses grupos, a variabilidade intraindividual geralmente foi maior para os pacientes com alteração na deglutição^(38)^ ou alguma doença de base específica^(6)^. Sabe-se que a variabilidade intra ou interindividual pode sofrer influência de fatores particulares do individuo como sexo, idade e diferenças anatomofuncionais. Quando além disso existe uma condição clínica adversa é necessário considerar que a dispersão dos dados pode ser maior em função das adaptações naturais do organismo.

A variabilidade pode ser um parâmetro importante para acompanhamento e detecção precoce de sinais e sintomas disfágicos. Em um estudo com manometria de alta resolução que investigou a pressão em diferentes regiões anatômicas durante o ato de deglutir foi visto que a variabilidade de pressão na velofaringe contribuiu para diferenciar pessoas saudáveis de pessoas com DP em estágio inicial a intermediário^(6)^. Além disso, verificou-se que para a avaliação da deglutição, uma abordagem com exames e protocolos complementares associados podem indicar alterações iniciais na DP que não são observadas em avaliações isoladas.

Um outro exemplo de estudo em pessoas com DP usou a eletromiografia de superfície^(24)^. Considerando a população com DP e níveis baixos de comprometimento da deglutição, foi encontrada uma diferença significativa na variabilidade da amplitude do pico eletromiográfico na região laríngea entre o lado mais afetado e o lado menos afetado pela doença na condição de deglutição de líquido fino. Os autores do estudo acreditam que a menor variação do lado mais afetado pode ser resultado da redução na produção de força na musculatura envolvida no ato de deglutir.

A maioria dos desfechos apontou para a influência do volume deglutido. Em um dos estudos que usou a videofluoroscopia, por exemplo, o CV foi mais alto na tarefa de deglutição de semissólido na maioria das medidas investigadas^(36)^. No outro estudo com videofluoroscopia foi realizada a análise dos escores da escala de aspiração de penetração (PAS)^(42)^. Os dados demonstraram alta variabilidade para as consistências de 20 ml líquido fino, 5 ml de líquido levemente espessado e de 3 ml líquido extremamente espessado. Os autores deste último estudo atribuem essa alta variabilidade à complexidade que os volumes maiores exigem do mecanismo de deglutição, bem como, à necessidade de deglutições múltiplas nas consistências mais espessas.

Averiguar a variabilidade dos parâmetros torna-se importante para compreender quais deles apresentam melhor homogeneidade diante das repetições analisadas, logo, quais são os mais confiáveis para avaliação e interpretação do desempenho da função investigada. Pode-se citar como exemplo um estudo que utilizou eletromiografia de superfície, no qual dentre as diversas medidas analisadas, a latência do *offset*, a duração e a amplitude máxima da atividade eletromiográfica durante a deglutição foram os parâmetros com menor variabilidade intraindividual^(31)^. Outro estudo revelou que a variabilidade ajudou a identificar que um método de deglutição voluntariamente modificável com oferta de instruções pode ser o mais indicado para a aplicabilidade da eletromiografia de superfície na prática clínica^(30)^.

Apesar disso, devido à alta dispersão intraindividual, verifica-se que é desafiador determinar valores que representem a faixa de normalidade esperada dos parâmetros quantitativos investigados. Um exemplo dessa complexidade é um estudo que usou manometria de alta resolução e nele foi verificado que a motilidade faríngea gerou pressões com graus de variabilidade diferentes conforme a região anatômica^(26)^.

Os distintos resultados do CV encontrados em cada artigo refletem a heterogeneidade dos métodos adotados nos estudos. Os artigos se preocuparam em usar o CV para verificar a variabilidade intraindividual ao repetir diferentes tarefas de deglutição pelo menos duas vezes e comparar os CV intraindividuais entre grupos distintos, seja por idade, condição clínica, volumes ou consistências alimentares.

Algumas limitações foram encontradas durante este mapeamento da literatura. O número de artigos elegíveis foi mais reduzido porque existem pesquisadores que investigaram a variabilidade por meio de medidas diferentes do CV ou utilizaram esse coeficiente para avaliar morfometria, força, resistência, fluxo respiratório ou de tosse, que embora sejam características associadas à deglutição, não avaliam necessariamente o indivíduo durante a execução da função. A heterogeneidade dos métodos restringiu a comparação entre os estudos e indicou a necessidade de maior padronização dos procedimentos de coleta e análise, incluindo o ponto de corte para interpretação do CV.

## CONCLUSÃO

Os estudos que analisaram a variabilidade de parâmetros quantitativos da deglutição obtidos por meio de exames instrumentais são heterogêneos e indicaram que a dispersão das medidas oscila entre baixa e alta de acordo com o tipo de exame, parâmetro investigado, presença ou não de doença de base e características do bolo alimentar como consistência e volume.
